# Hemodynamic and Uterotonic Effects of Carbetocin Versus Oxytocin in a Cesarean Section With a High Risk of Postpartum Hemorrhage

**DOI:** 10.7759/cureus.83504

**Published:** 2025-05-05

**Authors:** Vanshika Goel, Urmila Karya, Anupam Rani, Manvi Dayal

**Affiliations:** 1 Obstetrics and Gynaecology, Lala Lajpat Rai Memorial Medical College, Meerut, IND

**Keywords:** carbetocin, cesarean section, maternal morbidity, oxytocin, postpartum hemorrhage, uterotonic agents

## Abstract

Introduction: Postpartum hemorrhage (PPH) is a leading cause of maternal morbidity and mortality worldwide, particularly in developing countries. Effective uterotonic agents are crucial for minimizing blood loss and maintaining hemodynamic stability during deliveries. Carbetocin has emerged as a promising alternative to oxytocin for preventing PPH, especially in high-risk pregnancies.

Aims and objectives: This study aimed to compare the efficacy of carbetocin and oxytocin in controlling intraoperative blood loss, maintaining uterine tone, and reducing the need for additional uterotonics in women undergoing cesarean section (CS) with a high risk of PPH.

Materials and methods: This prospective case-control study was conducted on 200 pregnant women at high risk for PPH undergoing CS after obtaining written informed consent. Participants were randomly assigned to two groups. Group I (n = 100) received injection carbetocin (100 µg IV), while Group II (n = 100) received injection oxytocin (10 IU IV in 500 mL normal saline). The hemodynamic parameters of the participants, uterine tone, blood loss, and the need for additional uterotonics were recorded and analyzed.

Results: Carbetocin significantly reduced intraoperative blood loss, with 81 participants (81%) in the carbetocin group experiencing blood loss less than 500 mL compared to 54 participants (54%) in the oxytocin group. The need for additional uterotonics was significantly lower in the carbetocin group (13% vs. 43%; n = 100). Uterine tone was better in the carbetocin group both intraoperatively and two hours postoperatively (p < 0.0004), and the postoperative fall in hemoglobin was also less compared to the oxytocin group. Hemodynamic stability was maintained better in the carbetocin group, with fewer fluctuations in blood pressure.

Conclusion: Carbetocin was found to be more effective than oxytocin in managing high-risk cesarean deliveries, offering better control over intraoperative blood loss, maintaining uterine tone, and reducing the need for additional uterotonics. Its prolonged uterotonic effect and stable hemodynamic profile make it a superior choice for PPH prevention in CSs.

## Introduction

Postpartum hemorrhage (PPH) is a significant global health issue and a leading cause of maternal morbidity and mortality especially in developing countries, particularly uterine atony. PPH is defined as blood loss exceeding 500 mL in vaginal delivery or 1000 mL following a cesarean section (CS) or any amount of blood loss that results in hemodynamic instability. PPH is categorized into two main types: primary and secondary. Primary PPH, accounting for about 80% of cases, occur within the first 24 hours after delivery [1]. The physiological response causing myometrial fibers to contract and retract is responsible for control of PPH [2]. However, when this mechanism fails, the consequences can be dire, leading to rapid exsanguination and potentially fatal outcomes for the mother. Secondary PPH occurs between 24 hours and six weeks postpartum, often due to retained products of conception, infection, or both. Even non-fatal PPH can lead to severe complications such as anemia, need for blood transfusions, Sheehan's syndrome (a form of pituitary infarction), coagulopathy, and organ damage due to shock and hypotension [3].

In 2015, according to the World Health Organization (WHO), hemorrhage was the cause of 34% of the estimated 275,000 maternal deaths worldwide, equating to over 10 deaths per hour due to excessive bleeding during childbirth [4]. Then, in 2017, PPH was responsible for approximately 68,500 deaths annually out of 295,000 maternal deaths, with 99.7% of these occurring in low-income countries. The incidence of PPH varies by region, ranging from 2.6% in Asia to 10.5% in Africa [5]. In India, data from the Sample Registration System (SRS) for 2018-2020 indicates a maternal mortality rate of 97 per 100,000 live births. The stark contrast in maternal death rates across different regions highlights the disparities in healthcare access and the socioeconomic divide. Notably, 99% of maternal deaths occur in developing countries, underscoring the urgent need for improved maternal healthcare in these regions. In such cases, immediate and effective management with uterotonic agents is crucial to control blood loss, prevent shock and reduce the need for invasive interventions. Prophylactic uterotonic drugs and active management during the third stage of labor are essential strategies to prevent PPH and associated maternal mortality, emphasizing the importance of preserving maternal health and reducing delivery-related complications [6].

Several factors heighten the risk of PPH, including maternal anemia, thrombocytopenia, prolonged labor, placental abnormalities, multifetal gestation, preeclampsia, and advanced maternal age [7]. Previous PPH, previous cesarean deliveries, inherited hemostatic disorders, and a history of such conditions also increase risk. Notably, over 40% of PPH cases occur in women with no known risk factors, emphasizing the need for vigilant monitoring of all pregnant women. A multidisciplinary approach, including close monitoring of symptoms and vital signs, is essential for timely identification and intervention. Establishing a global consensus on PPH management is crucial for improving maternal health outcomes [8].

The guidelines on PPH issued by the Society of Obstetricians and Gynaecologists of Canada (SOGC) advocate for active management of the third stage of labor over expectant management and the administration of uterotonic drug is considered a fundamental component of active management protocols in preventing PPH by markedly decreasing its incidence. [9] Among these, oxytocin (10 IU administered intramuscularly) is favored for the prevention of PPH in low-risk vaginal and caesarean deliveries. Oxytocin binds to oxytocin receptors in the myometrium, stimulating the contraction of uterine smooth muscle. It exhibits a rapid onset but a brief duration.

An acceptable alternative for the management of PPH is the intravenous infusion of oxytocin (20 to 40 IU per 1000 mL, with a rate of 150 mL per hour). If it fails to control PPH, then, other agents such as carboprost, misoprostol, and methylergotamine derivatives are used as second line but most are associated with side effects. The use of ergometrine, previously employed in combination with oxytocin as syntometrine, was associated with a small reduction in the risk of PPH. It has been discontinued due to the increased risk of adverse maternal effects [10]. Oxytocin, a synthetic version of the natural hormone, has been the gold standard for PPH prevention. However, its short half-life necessitates continuous administration or repeated doses to maintain uterine tone. Other drawback of oxytocin is that it requires cold chain maintenance to preserve its efficacy which is difficult in low- and middle-income countries with warm climates [11].

To overcome these problems, carbetocin, a synthetic long-acting analogue of oxytocin, has recently been introduced as a more potent alternative without the need for continuous infusion and does not need cold chain maintenance. It exhibits a half-life approximately 40 minutes, which is significantly longer than that of oxytocin. Administration of the optimal dosage of 100 micrograms results in uterine contractions occurring within less than two minutes [12]. Given the importance of minimizing blood loss and ensuring maternal hemodynamic stability during CSs in high-risk populations, this study investigated the comparative effectiveness of carbetocin and oxytocin.

## Materials and methods

Study design and setting

The study design was a prospective case-control study, carried out at the Department of Obstetrics and Gynaecology, Lala Lajpat Rai Memorial (LLRM) Medical College, Meerut, North India. The study conducted from October 2022 to March 2024. Approval for the study was obtained from the LLRM Medical College Institutional Ethics Committee (approval no. SC-1/2024/5578).

Inclusion criteria

All women planned for cesarean delivery and willing to participate in the study with a high risk of PPH, such as multiple pregnancy, previous CS, placenta previa, presence of uterine fibroid, previous myomectomy, polyhydramnios, macrosomia, and previous history of PPH, were included.

Exclusion criteria

Patients with hypertension, preeclampsia, cardiac disease, renal disease, liver disease, epilepsy, need for general anesthesia, and history of hypersensitivity to carbetocin were excluded.

Procedure

Participants fulfilling the inclusion criteria were randomly allocated in two groups using a sealed paper envelop. Group I (carbetocin group) received a single intravenous bolus of 100 µg carbetocin and Group II (oxytocin group) received a 10 IU oxytocin infusion in 500 mL of normal saline immediately after the delivery of the baby.

Cesarean delivery was performed under spinal anaesthesia by a team of expert obstetricians. To evaluate the hemodynamic effects of carbetocin and oxytocin, BP (blood pressure) noted after spinal anaesthesia, one minute, three minutes, and five minutes after uterotonic drug administration, after uterine repair, and at the end of the caesarean procedure. The occurrence of any side effects of drugs such as nausea, vomiting, flushing, shivering, headache, dyspnea, and tachycardia were also recorded. The estimation of blood loss was assessed post-uterine incision using a separate suction machine for liquor and blood and by weighing blood and blood-soaked mops. The surgeon requested additional uterotonic agents on the basis of clinical findings during surgery. Postpartum blood loss during the first 24 hours after the operation was assessed by weighing blood-soaked napkins and clots if any. Hemoglobin and hematocrit were measured before surgery and 24 hours after the surgery to note drop in hemoglobin and hematocrit level. Uterine tone (standardized as Very good, Good, Sufficient, Atony) was monitored clinically intraoperatively at two hours, 12 hours, and 24 hours after surgery by a blinded observer who was a senior obstetrician to minimize bias. Urine output was monitored to assess the diuretic effect of the drug. Adverse effects of both drugs were evaluated up to 24 hours after delivery. 

PPH was considered by blood loss more than 1000 ml. Diagnosed PPH cases were treated with repeat oxytocin, misoprostol, methyl ergometrine, or carboprost and were taken as subjects treated with additional uterotonic.

Blinding

The operative team, observer, and participants were blinded to the group allotted to avoid bias. Only anesthetists were given the sealed envelope as to which drug to be used and they were not blinded.

Statistical analysis

The data generated was analyzed using IBM SPSS Statistics for Windows, version 20.0 (released 2011, IBM Corp., Armonk, NY). Continuous variables were represented as "mean (SD)" and categorical variables were represented as "frequency (percentage)." A chi-square test or Fisher’s exact test was used to assess differences in categorical data. Student's unpaired T-test was used for differences in the means of two independent data. There were cross-tabulations and correlations to explore the relationship. A p-value <0.05 is considered significant at a confidence interval of 95%.

## Results

The baseline demographic characteristics, including age, parity, gestational age, and the presence of risk factors for PPH, were comparable between the two groups (p > 0.05) (Table 1).

**Table 1:** Baseline characteristics of the patients in the carbetocin and oxytocin groups

Characteristics	Carbetocin group (n = 100)	Oxytocin group (n = 100)	P-value
Mean maternal age (years)	28.03 ± 3.74	7.81 ± 2.99	0.6464
Mean gestational age (weeks)	38.29 ± 2.08	38.04 ± 2.01	0.3885
Gravida distribution			0.1953
G1	5	5	
G2	43	43	
G3	23	34	
G4 and above	29	18	
Risk factors for PPH			0.0770
Previous 1 CS	61	63	
Previous 2 CS and above	18	27	
Antepartum hemorrhage	6	6	
Multifetal pregnancy	7	4	
Pregnancy with fibroids	4	0	
Polyhydramnios	2	0	
Previous history of PPH	2	0	

In the present study, it was observed that in both groups, the most common risk factor for PPH was previous 1 CS (61 participants (61%) in the carbetocin group and 63 participants (63%) in the oxytocin group), followed by previous 2 CS (18 participants (18%) in the carbetocin group and 27 participants (27%) in the oxytocin group (p-value 0.0770).

**Figure 1:**
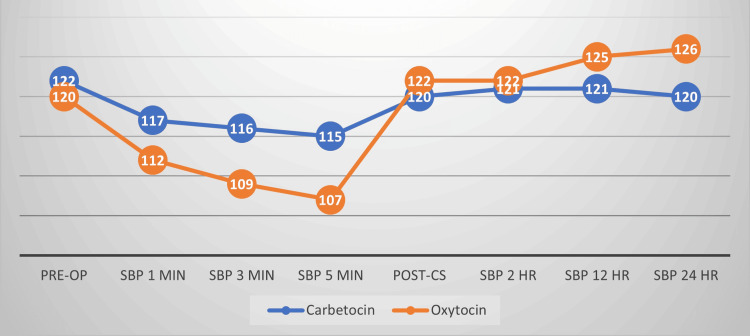
Systolic blood pressure change during and after the cesarean section

Both drugs have hypotensive effects after their administration, but oxytocin has more hypotensive effects as compared to carbetocin, as shown in Table 2. Systolic blood pressure (SBP) fell more in the oxytocin group as compared to carbetocin. After CS, SBP remains more stable in the carbetocin group, as shown in Figure 1. Similar findings were observed in regard to diastolic blood pressure change (Table 2), which was significant. Blood pressure fluctuation was less in the carbetocin group as compared to the oxytocin group, intra- and postoperatively, as shown in Table 2 and Figure 1.

**Table 2:** Comparison of the hemodynamic parameters

Parameter	SBP (systolic blood pressure) (mm Hg)		p-value	DBP (diastolic blood pressure) (mm Hg)		p-value
	Carbetocin	Oxytocin		Carbetocin	Oxytocin	
Preoperative	122.1 ± 9.49	120.66 ± 5.38	0.1887	76.56 ± 7.12	74.02 ± 3.38	0.0015
After 1 min. of uterotonic injection	117.46 ± 9.14	112.24 ± 5.52	<0.001	72.02 ± 6.68	66.94 ± 3.78	<0.001
After 3 min. of uterotonic injection	116.16 ± 8.52	109.9 ± 5.52	<0.001	70.8 ± 6.31	64.48 ± 3.98	<0.001
After 5 min. of uterotonic injection	115.94 ± 8.72	107.94 ± 4.94	<0.001	70.2 ± 6.19	62.62 ± 3.53	<0.001
Immediately after the cesarean section (CS)	120.78 ± 7.53	121.98 ± 3.41	0.0821	76.02 ± 4.57	76.04 ± 2.62	0.4851
Two hours postoperative	121.72 ± 8.25	122.72 ± 4.54	0.2899	76.8 ± 6.99	77.6 ± 3.21	0.2996
12 hours postoperative	121.58 ± 8.71	125.38 ± 4.26	<0.001	77.34 ± 5.54	79.48 ± 3.44	<0.001
24 hours postoperative	120.98 ± 8.81	126.66 ± 3.76	<0.001	76.48 ± 6.31	80.14 ± 3.46	<0.001

**Figure 2:**
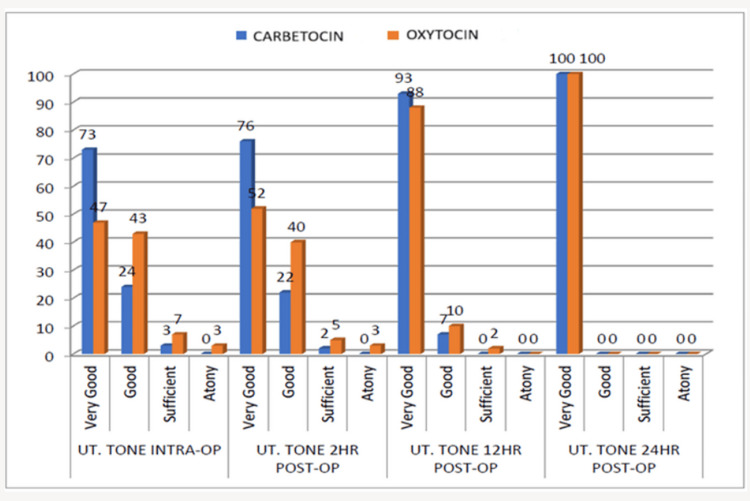
Uterine tone assessment during and after a cesarean section

Better uterine tone was observed in the carbetocin group during both the intraoperative and postoperative periods, with fewer cases of uterine atony when compared to the oxytocin group (Figure 2).

The mean intraoperative blood loss was significantly lower in the carbetocin group; 81 patients (81%) in the carbetocin group had blood loss <500 mL compared to 54 (54%) in the oxytocin group (p-value < 0.001). Postoperatively, both drugs showed similar results in terms of blood loss (p-value > 0.05) (Table 3).

**Table 3:** Maternal blood loss CS: cesarean section

Parameter	Group I (n = 100) carbetocin		Group II (n = 100) oxytocin		p-value
Blood loss CS	N=100	%	N=100	%	
<500 ml	81	81	54	54	<0.001
500-1000 ml	18	18	34	34	
>1000 ml	1	1	12	12	
Blood loss two hours post-op					0.0905
<500 ml	99	99	96	96	
500-1000 ml	1	1	04	04	
>1000 ml	0	0	0	0	
Blood loss 12 hours post-op					1.0
<500 ml	100	100	100	100	
500-1000 ml	0	0	0	0	
>1000 ml	0	0	0	0	
Blood loss24 hours post-op					1.0
<500 ml	100	100	100	100	
500-1000 ml	0	0	0	0	
>1000 ml	0	0	0	0	

The need for additional uterotonics was significantly lower in the carbetocin group, i.e., only 13 participants (13%) compared to the oxytocin group, 43 participants (43%), p < 0.01). In most cases, additional uterotonics in the oxytocin group were misoprostol tablet or oxytocin infusion.

In our study, drop in hemoglobin and hematocrit 24 hours after the CS was more in the oxytocin group compared to the carbetocin group (0.95 ± 0.40 g/dl vs. 1.39 ± 0.25 g/dl, p-value = 0.000; 3.33 ± 1.59 % vs. 6.6 ± 1.27, p-value = 0.000) (Table 4).

**Table 4:** Hemoglobin and hematocrit before and after a cesarean section CS: cesarean section

Parameter	Carbetocin (n = 100)	Oxytocin (n = 100)	p-value
Preoperative			
Hemoglobin (Hb) g/dl	9.93 ± 1.44	10.09 ± 0.85	0.1658
Hematocrit (Hct) %	31.99 ± 5.02	32.88 ± 3.83	0.0796
Postoperative drop in Hb and Hct			
Hb 24 hours after CS (g/dl)	0.95 ± 0.40	1.39 ± 0.25	<0.001
Hct 24 hours after CS (%)	3.33 ± 1.59	6.6 ± 1.27	<0.001

**Figure 3:**
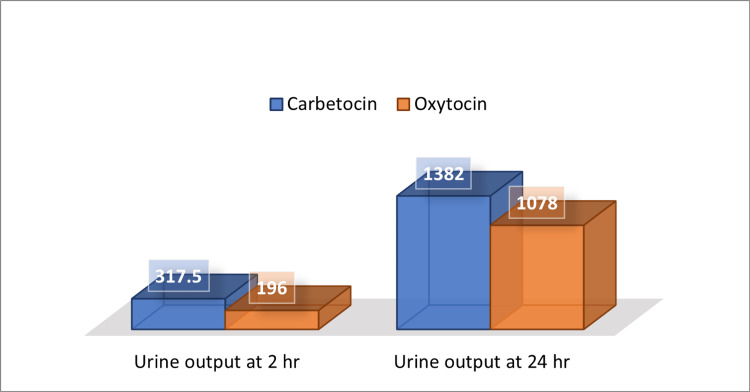
Urine output

The carbetocin group had a significantly higher mean urine output compared to the oxytocin group at two and 12 hours postoperatively, and the difference was statistically significant (p-value < 0.05), as shown in Figure 3.

It was also observed that a smaller number of participants required blood transfusion in the carbetocin group compared to the oxytocin group (5% vs. 17%, p-value = 0.0033).

The carbetocin group, 21 participants (21%) exhibited more adverse effects compared to the oxytocin group (six participants, 6%), with a p-value of 0.0012, which is statistically significant. Nausea and vomiting were the most common adverse effect in the carbetocin group (eight, 8%), followed by tachycardia (seven, 7%), dyspnea (two, 2%), headache (two, 2%), shivering (one, 1%), and flushing (one, 1%). In the oxytocin group, headache and tachycardia each accounted for 2% (two), while dyspnea and vomiting were noted in 1% (one) of cases.

## Discussion

PPH remains a significant cause of maternal mortality worldwide. Despite advancements in obstetric care, PPH still accounts for approximately 25% of maternal deaths globally, with the burden disproportionately higher in low-resource settings. Uterotonic agents are crucial in managing and preventing PPH by promoting uterine contractions and minimizing blood loss [13].

One of the most critical outcomes in the management of PPH is controlling intraoperative blood loss. The results of this study indicate that carbetocin offers significant advantages over oxytocin in terms of reducing intraoperative blood loss, maintaining uterine tone, and promoting hemodynamic stability.

Carbetocin's ability to maintain effective uterine tone without the need for continuous infusion is a significant clinical advantage, especially in resource-limited settings where continuous monitoring and infusion might not always be feasible. The need for fewer repeat doses or additional uterotonics also reduces the risk of side effects associated with high dose or prolonged use of oxytocin.

Hemodynamic stability is a crucial consideration in the management of patients undergoing CSs, particularly those at high risk for PPH. Uterotonics such as oxytocin are known to cause hemodynamic side effects, including hypotension and tachycardia, due to their vasodilatory effects. In our study, carbetocin demonstrated superior hemodynamic stability compared to oxytocin.

The longer half-life of carbetocin (40 minutes vs. 10-15minutes for oxytocin) likely contributes to its prolonged uterotonic effect, resulting in better sustained uterine contractions and reduced blood loss. This is particularly beneficial in CSs, where uterine atony is more likely to occur due to the surgical manipulation of the uterus.

Larciprete et al. (2013) [14] and Moertl et al. (2011) [15] also reported that carbetocin had fewer adverse hemodynamic effects compared to oxytocin. The vasodilatory effects of oxytocin are known to cause abrupt drops in blood pressure, especially when administered as a rapid bolus. By contrast, carbetocin’s slower onset and longer duration of action likely contribute to its more stable hemodynamic profile, making it a safer choice.

Our study found that women in the carbetocin group had better uterine tone intraoperatively and postoperatively compared to those in the oxytocin group. The maintenance of uterine tone after delivery is a key factor in preventing PPH. This finding is supported by studies from Rath Werner (2009) [16] and Attilakos et al. (2010) [17], which reported that carbetocin induces more sustained uterine contractions compared to oxytocin. The ability of carbetocin to maintain uterine tone over a prolonged period may be due to its longer half-life and oxytocin receptor affinity, which ensures continuous uterine contraction and reduces the risk of uterine atony.

We observed a significant decrease in the need of blood transfusion and less fall in hemoglobin and hematocrit postoperatively in the carbetocin group. This is highly advantageous for countries like India, where anemia in pregnancy is a major health concern, as using carbetocin in high-risk cases can lead to decreased post CS anemia and blood transfusion. As a result, it can overall decrease maternal morbidity and mortality. 

One of the significant outcomes in this study was the reduced need for additional uterotonic agents in the carbetocin group. (13% (13) in the carbetocin group vs. 43% (43) in the oxytocin group). This finding reinforces carbetocin’s superiority over oxytocin to sustain uterine contractions and prevent uterine atony.

The need for additional uterotonics, such as misoprostol, ergometrine, and oxytocin infusion in higher doses, increases the risk of side effects, which can complicate the management of PPH. Carbetocin’s prolonged action minimizes the need for additional interventions, which is particularly important in settings where access to a variety of uterotonic agents is limited.

The findings of this study have significant clinical implications in the management of PPH, particularly in high-risk CSs. Carbetocin’s ability to provide sustained uterine contraction, reduce blood loss, and maintain hemodynamic stability makes it a valuable tool in obstetric practice. Its single-dose administration also simplifies PPH prevention, reducing the need for continuous infusion or repeat dosing, which is particularly useful in low-resource settings.

However, despite these advantages, the cost of carbetocin remains higher than oxytocin, which may limit its widespread use, particularly in low-resource settings. Future research should focus on cost-benefit analyses to determine whether the improved clinical outcomes associated with carbetocin justify its higher cost in different healthcare environments.

The strength of this study is practical application and potential impact on maternal health. This study can contribute in improving maternal health by significantly decreasing PPH and its potential ill effects. This study could contribute in future policy making for management of PPH.

While this study provides valuable insights into the comparative efficacy of carbetocin and oxytocin, there are some limitations that should be considered. First, the study was conducted in a single center, which may limit the generalizability of the findings to other populations or healthcare settings. A multicenter study involving a more diverse population could provide more comprehensive results.

## Conclusions

The present study demonstrates that carbetocin when compared to oxytocin is a more effective uterotonic agent in managing PPH in high-risk CSs. Carbetocin offers better control over intraoperative blood loss; superior uterine tone, reducing the need for additional uterotonics; and improved hemodynamic stability. Given its prolonged action and favorable safety profile, carbetocin should be considered as the first-line uterotonic agent for PPH prevention in high-risk populations. However, further studies are needed to assess its long-term efficacy and cost-effectiveness in different clinical settings.
